# Impact of vaccination delay on deaths averted by pneumococcal conjugate vaccine: Modeled effects in 8 country scenarios

**DOI:** 10.1016/j.vaccine.2019.07.063

**Published:** 2019-08-23

**Authors:** Emily D. Carter, Yvonne Tam, Neff Walker

**Affiliations:** Institute for International Programs, Johns Hopkins Bloomberg School of Public Health, 615 North Wolfe Street, Baltimore, MD, USA

**Keywords:** Pneumococcal conjugate vaccine, Vaccination delay, Vaccination, Timing, Child mortality, Mathematical model

## Abstract

Delay in vaccination from schedule has been frequently documented and varies by vaccine, dose, and setting. Vaccination delay may result in the failure to prevent deaths that would have been averted by on-schedule vaccination.

We constructed a model to assess the impact of delay in vaccination with pneumococcal conjugate vaccine (PCV) on under-five mortality. The model accounted for the week of age-specific risk of pneumococcal mortality, direct effect of vaccination, and herd protection. For each model run, a cohort of children were exposed to the risk of mortality and protective effect of PCV for each week of age from birth to age five. The model was run with and without vaccination delay and difference in number of deaths averted was calculated. We applied the model to eight country-specific vaccination scenarios, reflecting variations in observed vaccination delay, PCV coverage, herd effect, mortality risk, and vaccination schedule. As PCV is currently being scaled up in India, we additionally evaluated the impact of vaccination delay in India under various delay scenarios and coverage levels.

We found deaths averted by PCV with and without delay to be comparable in all of the country scenarios when accounting for herd protection. In India, the greatest relative difference in deaths averted was observed at low coverage levels and greatest absolute difference was observed around 60% vaccination coverage. Under moderate delay scenarios, vaccination delay had modest impact on deaths averted by PCV in India across levels of coverage or vaccination schedule. Without accounting for herd protection, vaccination delay resulted in much greater failure to avert deaths.

Our model suggests that realistic vaccination delay has a minimal impact on the number of deaths averted by PCV when accounting for herd effect. High population coverage can largely over-ride the deleterious effect of vaccination delay through herd protection.

## 1 Introduction

Vaccines prevent 2–3 million deaths each year [1]. A high proportion of vaccine-preventable deaths occur in early infancy. While some deaths cannot be directly prevented because of the minimum age of vaccination, delay in vaccination from schedule can result in preventable deaths. Numerous studies have documented delays in vaccination in multiple settings [2], [3], [4], [5], [6], [7], [8]. Evidence from Sanderson and colleagues suggest delay in vaccination is common, although the degree and frequency of delay varies greatly by vaccine, dose, and country. Median vaccination delay across low- and middle-income countries (LMICs) in 50% coverage ranged from two weeks for bacille Calmette-Guerin (BCG) to six weeks for the third dose of diphtheria-tetanus-pertussis (DTP), with a handful of countries exhibiting much more extreme delays [2]. However, the impact of vaccination delay on child mortality has not been addressed. It is important to understand the effect of vaccination delay on mortality to ensure correct estimation of vaccine impact and guide efforts to maximize the health impact of vaccination programs.

*Streptococcus pneumoniae* is a major cause of pneumonia, as well as meningitis and bacteremia. Pneumonia is a leading global cause of child deaths. Prior to the introduction of pneumococcal conjugate vaccine (PCV) in 2000, *S. pneumoniae* was responsible for more 800,000 deaths in children 1–59 months annually [9]. Most deaths occur in children under two years of age, with approximately half of deaths occurring in infants [9], [10]. PCV targets the most common of the 90+ pneumococcal serotypes; the 10-valent and 13-valent formulations have largely replaced the older 7-valent formulation [11]. PCV is efficacious in preventing 80% of vaccine-serotype invasive pneumococcal disease (IPD) [12]. PCV is commonly administered from six weeks of age on either a two or three primary dose schedule, with or without a booster dose around one year of age. Some country schedules delay the first dose be given at two months rather than six weeks of age [13]. PCV has been introduced in over 130 countries and global vaccination coverage was estimated at around 42% in 2016 [14].

Delay in vaccination from recommended vaccination schedule may reduce the protective impact of vaccination on child mortality, particularly if the age period between vaccination schedule and vaccine receipt corresponds to a period of high vaccine-preventable mortality risk. Due to the peak in pneumococcal deaths in early childhood, delay in vaccination with PCV can result in additional vaccine-preventable deaths. We explored the effect of PCV vaccination delay on *S. pneumoniae* mortality in children under five in various country-specific scenarios using a model for estimating the impact of vaccination on child mortality by fine age intervals.

## 2 Methods

Assessing the effect of vaccination delay on vaccine-preventable under-five mortality requires modeling the interplay of vaccination coverage and mortality risk by fine age intervals. We generated estimates of *S. pneumoniae*-specific mortality by week of age. We modeled vaccination coverage by week of age to calculate the direct and indirect protective effect of PCV by week of age. The model allows exposure of a cohort of children to the weekly mortality risk and protective effect of PCV to calculate the number of deaths prevented by PCV under different vaccination timing scenarios and country settings. The model was applied to eight country-specific scenarios using vaccination data from the Democratic Republic of the Congo (DRC), Ethiopia, India, Laos, Nigeria, Pakistan, Swaziland, and Zimbabwe.

**Fig. 1:**
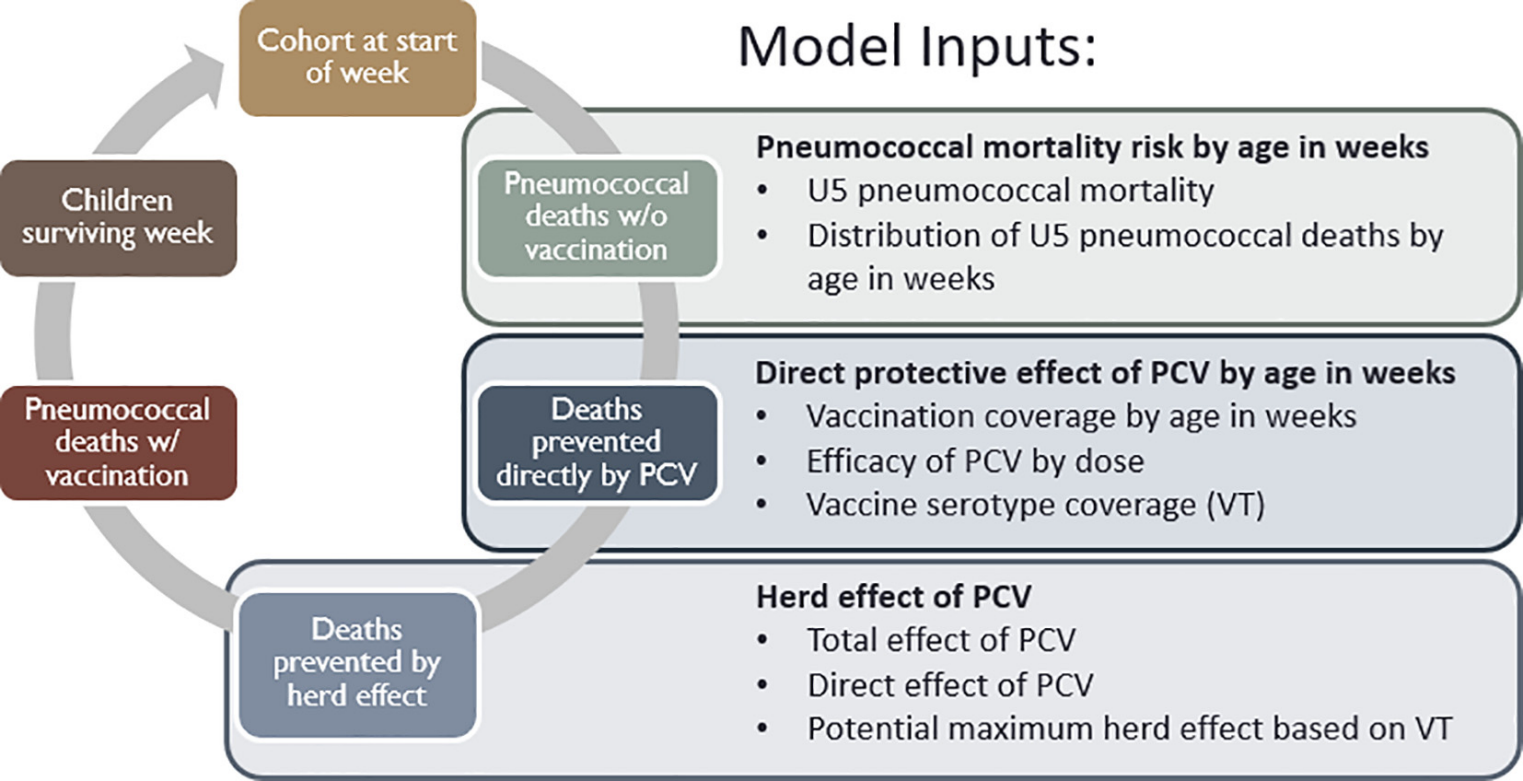
Model for calculating impact of PCV on child pneumococcal mortality.

The vaccine delay model was constructed as a deterministic mathematical model in Stata 14.2 (*StataCorp LLC, College* Station, Texas). The model functions by exposing an annual birth cohort of children to the week of age-specific risk of mortality from pneumococcal pneumonia and meningitis, and protective effect of PCV vaccination. Each model component was defined using parameter values derived from existing literature. For each week of age, the risk of mortality is estimated based on the age-specific distribution of pneumococcal deaths and the direct protective effect of PCV is estimated based on the age-specific vaccination coverage. The herd effect of PCV is constant across all ages and calculated based on the three-dose PCV coverage among children age 24–36 months. The birth cohort is (1) exposed to the week of age-specific risk of mortality, and pneumococcal deaths in the absence of vaccination are calculated, (2) the week of age-specific direct effect of PCV based on vaccination coverage is applied, and the number of deaths averted directly by vaccination are calculated, (3) the herd effect is applied to those children not directly protected by PCV, and the number of deaths averted indirectly by population vaccination coverage are calculated, (4) residual deaths are calculated and removed from the cohort, and (5) surviving children in the cohort are exposed to the next week of age risk and protective effect (Fig. 1). Inputs used in each step of the model are further described below. A detailed diagram of the model, including model inputs and their sources, model structure, and basic model processes, is given in supplemental Fig. 1.

### 2.1 Pneumococcal mortality by age in weeks

There are no existing estimates of *S. pneumoniae*-specific mortality or distribution of *S. pneumoniae* deaths by week or month of age. We generated estimates of week of age-specific under-five pneumococcal mortality rates using existing estimates of country-specific under-five mortality rate (U5MR) [15], the proportion of under-five deaths due to pneumonia and meningitis [16], the proportion of pneumonia [17] and meningitis deaths [18] due to S. *pneumoniae* in the absence of *Haemophilus influenzae* type B (Hib) or PCV vaccination, and the modeled distribution of IPD deaths. We modeled the age distribution of deaths from *S. pneumoniae* based on the binned distribution of IPD deaths reported by five studies with a sample of >25 IPD deaths included in a review by Russell and colleagues [10]. A gamma distribution was fit to the pooled data from the five studies. A gamma distribution was chosen to model the distribution of IPD deaths because Russell and colleagues reported a gamma distribution fit the empirical data on deaths by age better than alternative distributions [10]. The probability density function (PDF) of the gamma distribution of IPD deaths was modeled using Eq. (1).

(1) $$\mathrm{PDF}\;of\;IPD\;Deaths=0.0005+\frac{1}{\Gamma \left(k\right)\theta^{k}}x^{k-1}e^{-\frac{x}{\theta }};$$

where:

k = shape of gamma distribution = 1.7
Θ = scale of gamma distribution = 23
x = age in weeks

The distribution of IPD deaths within the age range of 0–59 months was then scaled to sum to 100% and the week-specific percentage of deaths was applied to distribute the estimate of total under-five pneumococcal deaths across the period by week of age.

### 2.2 Vaccination timing and coverage

The distribution of vaccination timing was defined using data from Clark and colleagues [19] developed using methods for calculating week of age vaccination coverage from Demographic and Health Survey (DHS) and Multiple Indicator Cluster Survey (MICS) vaccination data as outlined in their 2009 publication [2]. DHS and MICS do not typically capture data specifically on PCV. However, DTP vaccination is captured and in our selected countries the PCV and DTP schedule and final vaccination coverage, as estimated by WHO-UNICEF [20], align suggesting DTP vaccination timing is a reasonable proxy for PCV timing.

Dose-specific cumulative vaccination coverage was modeled as the cumulative distribution function (CDF) of an exponential distribution fit based on the country-specific median delay from schedule in vaccine receipt. We modeled the CDF of the cumulative percentage of final vaccination coverage by age in weeks with a rate parameter defined by median vaccination delay from schedule among children vaccinated by three years using Eq. (2). All early vaccination (prior to schedule) was modeled as vaccination on the schedule date.

(2) $$Cumulative\;Dose-Specific\;Vaccination=1-e^{-\lambda x};$$

where:

▪ $\lambda =\frac{ln(2)}{\mathrm{Median}\;age\;dose\;received-dose\;schedule}$
▪ x = Age in weeks − dose schedule

**Fig. 2:**
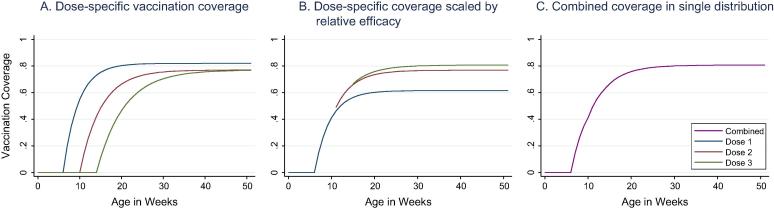
Model of vaccination coverage by week in age in DRC.

Timing of each dose was modeled independently and then combined into a single distribution, scaled by the relative dose-specific efficacy of PCV as shown in Fig. 2 [21]. Coverage of the second and third doses are conditional on receipt of the first and second dose respectively. The coverage of PCV dose two and three were modeled as additive protection above PCV dose one through improved vaccine efficacy.

### 2.3 Direct effect

The proportion of children directly protected at each week of age was calculated based on proportion of children vaccinated and the efficacy of the vaccine. The week of age-specific direct protective effect of vaccination was calculated by applying the three-dose vaccine-type efficacy of PCV [12] and region and formulation vaccine-serotype coverage [22] to the scaled cumulative vaccination coverage.

### 2.4 Herd effect

Herd effect was included in the model as an additional proportion of vaccine-susceptible pneumococcal deaths that could be prevented by population-level PCV coverage beyond those deaths prevented directly by vaccination. The total protective effect of PCV on IPD was modeled by Liu and colleagues [23] based on data from a study by Guevara [24]. Liu’s model assumed no herd effect if vaccination coverage was below 13% and capped the total effect of PCV at the vaccine serotype coverage, based on regional estimates of the proportion of IPD cases caused by vaccine-type (VT) serotypes [22]. The herd effect of PCV was modeled as the total effect of PCV on vaccine-susceptible pneumococcal cases minus the direct effect of PCV on VT-IPD among vaccine-susceptible cases not directly protected by the vaccine using Eq. (3). Unlike our direct effect estimates which vary by week of age, the herd effect of reduced transmission due to population coverage was modeled as constant across child ages based on the final three-dose coverage of PCV.

(3) $$\mathrm{Herd}\;Effect=\left\{\begin{array}{cc} 0, & 3\;dose\;coverage<0.13\\ \frac{\left(\left(\%SpVT+0.31*\ln\left(3dosecoverage\right)\right)-\left(VE*3dosecoverage*\%SpVT\right)\right)}{\left(\%SpVT-\left(VE*3dosecoverage*\%SpVT\right)\right)}, & 3\;dose\;coverage\geq 0.13 \end{array}\right)$$

### 2.5 Model scenarios

We applied the model using eight country-specific vaccination scenarios. We selected the countries to reflect a diverse range of vaccination scenarios and a significant proportion of global child pneumonia mortality. Together, India, Pakistan, Nigeria, DRC, and Ethiopia account for almost half of all global child pneumonia deaths [25]. Swaziland was selected as a country with very little vaccination delay, while Zimbabwe and Laos were selected for having long vaccination delays. Country scenarios also varied by underlying population at risk, three-dose PCV coverage, and vaccination schedule (Table 1). For each country scenario, excluding India, we ran the model using the country-specific median vaccination delay from schedule, calculated as described previously [19], and documented PVC coverage [20]. Each scenario was run using the country-specific inputs on annual births [26], under-five mortality [15], proportion of cases susceptible to PCV [22], and vaccination schedule [13], in addition to country-specific vaccination coverage and delay.

**Table 1:** Country-specific vaccination scenario inputs.

Country	Births in 2016	U5MR in 2016	% Cause of death from pneumonia/Meningitis	% VT	Schedule and median delay	3 Dose coverage in 2016
Ethiopia	3,081,883	58.4/1000	16.4/2.1	77	Schedule 6/10/14 wks +3.5/4.5/5.5 wks	76

Nigeria	6,682,764	104.3/1000	19/2	77	Schedule 6/10/14 wks +2.5/3.5/5.5 wks	26

DRC	3,091,606	94.3/1000	16.1/2.5	77	Schedule 6/10/14 wks +1.5/2.5/3.5 wks	77

Pakistan	5,190,882	78.8/1000	14.8/1.1	74	Schedule 6/10/14 wks +1.5/+2.5/4.5 wks	72

Swaziland	38,797	70.4/1000	15.7/1.1	77	Schedule 6/10/14 wks +0/0/0.5 wks	90

Zimbabwe	525,793	56.4/1000	14.7/1.4	77	Schedule 6/10/14 wks +7.5/9.5/11.5 wks	90

Laos	162,943	63.9/1000	17.7/1.4	74	Schedule 6/10/14 wks +7.5/19.5/28 wks	78

India	24,736,159	43/1000	14.6/1.7	74	Schedule 6/14 wks + 9 months N/A	N/A

India was further selected to explore the impact of various vaccination timing scenarios. India is currently scaling up PCV in multiple states under a two primary dose schedule at six and 14 weeks and a booster dose at nine months. We modeled the impact of vaccination delay in India under various delay scenarios and coverage levels. We modeled the impact of vaccination delay over a range of final vaccination coverage levels ranging from 1% to 100% vaccination coverage. We developed five vaccination timing scenarios based on data from Clark and colleagues (Table 2) using vaccination timing data most closely corresponding to India’s 6/14 week plus 9-month schedule, specifically data on timing in countries with DTP dose one scheduled at six weeks, DTP dose three scheduled at 14 weeks, and measles vaccination at nine months. Across countries with this schedule, we identified both the shortest and longest delays, median delay, bottom quartile delay, and the delay observed in India. We also explored the effect of median and bottom quartile delays in India under alternative vaccination schedules, including 3 + 0 primary dose schedule with doses at 6/10/14 weeks and 2/4/6 months, and 2 + 1 schedule with doses at 2/4 months and a 12-months booster (Supplementary Table 1). Each of the timing scenarios with coverage ranging from 1 to 100% was run using the annual births [26], under-five mortality [15], and proportion of cases susceptible to PCV [22] in India.

**Table 2:** India PCV delay scenarios under current schedule.

Delay	Source	#Wks delay to 50% final coverage
2 + 1 Median delay	Global median delay in receipt of DTP1, DTP3, & MCV*	Schedule 6/14 weeks + 9 months +1.5/+4.5/+1.5 wks

2 + 1 Quartile delay	Bottom quartile delay in receipt of DTP1, DTP3, & MCV*	Schedule 6/14 weeks + 9 months +2.5/+7/+2.5 wks

2 + 1 India delay	India-specific delay in receipt of DTP1, DTP3, & MCV	Schedule 6/14 weeks + 9 months +2.5/+5.5/+2.5 wks

2 + 1 Short delay	Shortest observed delay in receipt of DTP1, DTP3, & MCV*	Schedule 6/14 weeks + 9 months +0/+0.5/+0 wks

2 + 1 Long delay	Longest observed delay in receipt of DTP1, DTP3, & MCV*	Schedule 6/14 weeks + 9 months +7.5/+28.5/+12.5 wks

* Among countries assessed by Clark et al. (updated December 2014).

Each scenario was run with and without vaccination delay. We calculated the number of deaths averted by PCV in each scenario and number of residual deaths. For each scenario we compared the number of deaths averted with and without a vaccination delay and calculated the absolute and relative difference in deaths averted. We also ran sensitivity analyses with no herd effect to assess the role of herd protection in moderating the effect of vaccination delay.

## 3 Results

Running the country-specific scenarios in the model, we found very little impact of vaccination delay on the absolute or relative number of deaths averted by PCV (Table 3). The greatest absolute difference in number of deaths averted was observed for Nigeria, where a moderate vaccination delay and low three-dose coverage resulted in 600 additional deaths equating to 3% of the total deaths averted by PCV. Laos, with the most extreme observed delay, had the greatest relative difference in deaths averted at 4.1% equating to an absolute difference of 21 deaths. The other five countries had an absolute difference of fewer than 200 deaths and less than 3% relative difference in numbers of deaths averted. Table 3 shows the number of deaths averted directly and indirectly by PCV. Under the delay model, a greater number of deaths were indirectly averted by PCV, compared to scenarios with all on-schedule vaccination (no delay).

**Table 3:** Absolute and relative differences in deaths averted under country scenarios.

				Delay		No delay			
Country	3 Dose coverage	Herd	Median delay in weeks	Deaths averted (Direct + Herd)	Deaths	Deaths averted (Direct + Herd)	Deaths	Absolute difference	Relative difference
Ethiopia	76%	72%	+3/4/5	7577 (4495 + 3082)	3834	7732 (5046 + 2686)	3679	155	2.00%

Nigeria	26%	32%	+2/3/5	18,620 (9563 + 9057)	31,111	19,220 (10439 + 8781)	30,514	600	3.12%

DRC	77%	73%	+1/2/3	12,983 (8285 + 4698)	6181	13,128 (8813 + 4315)	6037	145	1.10%

Pakistan	72%	68%	+1/2/4	13,779 (8303 + 5476)	8397	13,956 (8847 + 5109)	8221	177	1.27%

Swaziland	90%	85%	+0/0/0.5	113 (82 + 31)	41	113 (84 + 29)	41	0	0%

Zimbabwe	90%	85%	+7/9/11	1184 (730 + 454)	459	1207 (879 + 328)	436	23	1.09%

Laos	78%	72%	+7/19/28	425 (226 + 199)	253	446 (299 + 147)	233	21	4.07%

**Fig. 3:**
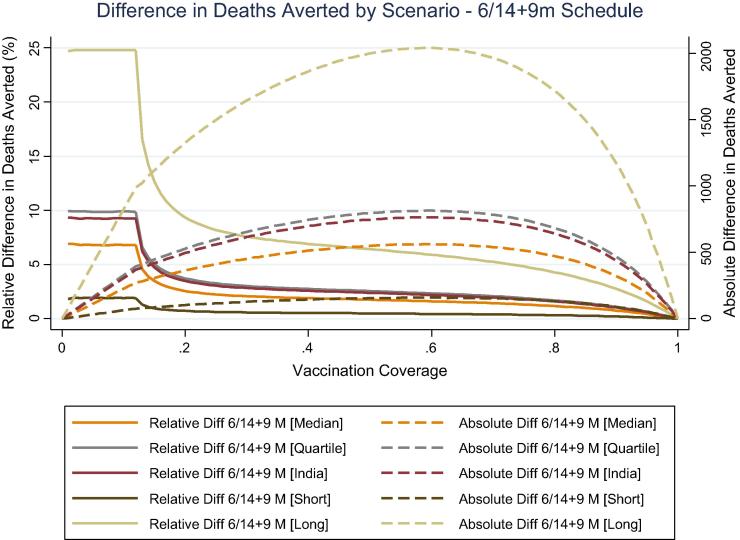
Absolute and relative difference in deaths averted in India under different delay scenarios and levels of vaccination coverage.

Applying the current PCV vaccination schedule in India with varying levels of coverage and delay scenarios, we found a wide range of differences in absolute and relative number of deaths averted (Fig. 3). Overall, we found that the greatest relative effect of delay on deaths averted occurred at low coverage levels (<13%) where the model assumed no herd effect. Under the most extreme delay scenario, simulating with the longest observed delay from Laos, the largest relative difference in number of deaths averted was approximately 25% and occurred at low levels of final vaccination coverage corresponding to an absolute difference in deaths fewer than 1000. Under more moderate delay scenarios, the relative difference in deaths averted was <10% and fell with increasing coverage levels consistent with increasing herd protection. With the most extreme delay scenario, the relative difference dropped to <10% when final vaccination coverage exceeded 20%. The largest absolute difference in deaths averted was observed around 60% three-dose vaccination coverage across scenarios, corresponding to a <6% relative difference in deaths averted in the most extreme scenario and <3% difference across all other scenarios. The most extreme delay scenario resulted in a peak absolute difference of 2040 deaths, however this represented only 5.9% of the total deaths averted (26,174) at 60% vaccination coverage. Using data on DTP and measles vaccination timing in India, the expected delay in receipt of PCV is moderate. Under the expected India-specific delay scenario, the greatest relative difference was 9.3% at <13% coverage, corresponding to an absolute difference of 370 deaths. The greatest absolute difference was 763 deaths, corresponding to a 2.2% relative difference in number of deaths averted at 58–60% coverage.

### 3.1 Alternative schedule

The impact of delay in India under alternative vaccination schedules was explored. Under the alternative schedules, similar results were observed to the results using India’s current schedule with moderate delays (Supplementary Fig. 2). The relative difference in deaths averted did not exceed 10% for any of the alternative schedules. The absolute difference did not exceed 700 deaths. Less of a difference in deaths averted was observed with later schedules.

### 3.2 Model without herd effect

Without accounting for herd effect, vaccination delay has a much greater impact on the number of deaths averted by PCV (Table 4). The largest difference comparing country-specific estimates with and without the herd effect was observed in scenarios with long delays and high three-dose vaccination coverage. In countries with the longest delay, Zimbabwe and Laos, the relative difference in deaths averted increased to 17% and 24.4% respectively. These estimates are 8.5 and 5 times greater than the estimates generated when accounting for herd effect. In Nigeria, where three-dose coverage was low and the delay was moderate, the estimated relative difference in deaths averted without herd protection was only 2.7 times greater than with a herd effect.

**Table 4:** Absolute and relative differences in deaths averted under country scenarios without herd protection.

				Delay		No Delay			
Country	3 Dose coverage	Herd	Median delay in weeks	Deaths averted (Direct + Herd)	Deaths	Deaths averted (Direct + Herd)	Deaths	Absolute difference	Relative difference
Ethiopia	76%	0%	+3/4/5	4492	6913	5044	6363	552	10.94%

Nigeria	26%	0%	+2/3/5	9555	40,141	10,432	39,279	877	8.41%

DRC	77%	0%	+1/2/3	8277	10,872	8806	10,345	529	6.01%

Pakistan	72%	0%	+1/2/4	8297	13,866	8842	13,324	545	6.16%

Swaziland	90%	0%	+0/0/0.5	82	72	84	70	2	2.38%

Zimbabwe	90%	0%	+7/9/11	730	913	879	764	149	16.95%

Laos	78%	0%	+7/19/28	226	452	299	380	73	24.41%

**Fig. 4:**
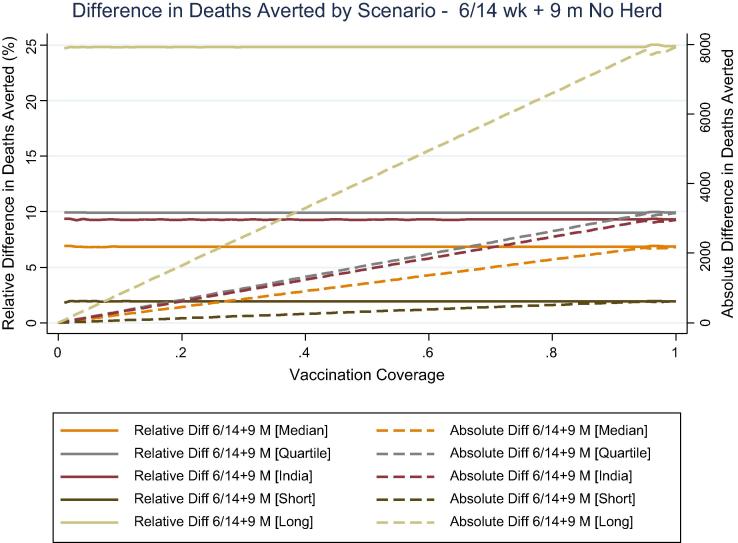
Absolute and relative difference in deaths averted in India under different delay scenarios and levels of vaccination coverage, without herd protection.

In India, the relative difference was roughly constant across coverage levels by scenario and was the same as that observed below 13% coverage in the model accounting for herd protection (Fig. 4). The absolute difference in deaths averted increased linearly with increasing coverage in the absence of a herd protection. This resulted in large absolute differences in deaths averted at high coverage levels, including a difference of over 8000 deaths with the most extreme delay scenario and 2182–3162 deaths with more moderate delay scenarios.

## 4 Discussion

Our model suggests that realistic vaccination delay has a minimal impact on the number of deaths averted by PCV when accounting for herd effect. In the seven country scenarios, observed vaccination delays resulted in small absolute and relative differences in the number of deaths averted by PCV. Under various vaccination delay and coverage scenarios in India, the greatest relative differences in deaths averted were observed at low final vaccination coverage levels where no herd effect was assumed. The greatest absolute differences were observed around 60% vaccination coverage but corresponded to small relative differences in deaths averted.

The model did not account for competing mortality risks, which would lead to children dropping out of the cohort because of non-pneumococcal mortality. Additionally, it did not account for in or out of country migration. The model also did not account for geographic distribution in vaccination and clustering of unvaccinated populations which could result in variable herd protection within a population. Limited data were available for some assumptions in the model, particularly around age distribution of pneumococcal deaths, herd effect of PCV at varying coverage levels, and dose-specific vaccine efficacy. The best available data were used, deferring to estimates that would yield greater effects of delay where assumptions were uncertain.

Empirical data were not available to externally validation our model of vaccination delay impact on child mortality. However, our model was derived using the structure and key model parameters employed in the Lives Saved Tool (LiST). Numerous analyses have demonstrated the validity of LiST modelled estimates of the impact of health intervention coverage on child mortality [27], [28], [29], [30]. LiST is one of multiple models that participate in the consortium for modeling vaccination impact for Gavi, the Vaccine Alliance [31], [32].

The degree of vaccination delay, overall vaccination coverage, and vaccination schedule affected the impact on deaths averted. Large delays in vaccination may result in notable relative differences in deaths averted in settings with low vaccination coverage, such as during the initial introduction of PCV. However, in low coverage situations the overall impact of PCV is minimal, resulting in small absolute differences in deaths averted. High coverage results in significant herd protection minimizing the deaths due to delay even under extreme delay scenarios. Moderate absolute differences in numbers of deaths averted may occur in countries where there is a large population at risk, moderate vaccination coverage, and a moderate to long delay in vaccination. However, these large absolute differences represent a small fraction of the total deaths averted by PCV under these conditions. Vaccination delay has less of an effect in countries with later vaccination schedules as the peak in mortality occurs prior to the vaccination schedule, and as a result the delay period occurs at a time of declining mortality rates.

Herd protection plays an important role in minimizing the effect of vaccination delay. In settings where there is moderate or high vaccination coverage, the herd effect protects a large proportion of unvaccinated children, including those infants that are delayed in receiving vaccination. The moderating effect of herd protection is clear in the estimates of numbers of deaths averted directly and indirectly by PCV under scenarios with and without a delay in vaccination receipt. In our models, a much greater impact of delay on deaths averted was observed when herd protection was removed from the model.

Further, models that do not account for herd protection may greatly underestimate the impact of vaccination on child mortality. Gavi, the Vaccine Alliance, tracks global progress through routine estimates of vaccines’ impact on child mortality [31], [33]. It is essential to accurately account for herd effect of vaccines in modeling impact on child mortality. Herd effect varies by vaccine and scenario, being driven by vaccination coverage and dispersion, vaccine efficacy, and aspect of the pathogen such as force of transmission [34], [35]. Vaccines for pathogens with high basic reproduction numbers, such as measles, do not display a significant herd effect until very high coverage is reached. However, for less transmissible pathogens herd effect is observed at more moderate coverage levels [36]. Under Gavi-supported vaccine introduction coverage is reached in just a few years’ time, further emphasizing the need to account for herd effects. Reduced nasal carriage has additionally contributed to significant herd effect observed with introduction of PCV and Hib vaccines [37]. While vaccination delay has minimal effects on estimates of vaccine impact, it remains an important issue for consideration in vaccination program planning and implementation. A number of factors are associated with delay in vaccination, including maternal education, migration, and lack of healthcare access and support [38], suggesting means of intervening to improve timely vaccination.

These results suggest overall vaccination coverage is a more important driver of vaccine mortality impact than vaccination timing. High population coverage can largely over-ride the deleterious effect of vaccination delay through herd protection. It is important to explore the impact of delay on child mortality for other vaccines, as the age distribution of pathogen-specific mortality and characteristics of the pathogen and vaccine tied to disease transmission and herd protection play important roles in driving the impact of delay on mortality. Efforts to increase vaccination coverage are paramount to achieving greater reductions in child mortality and thoughtful consideration should be given to how to best utilize resources.
