# Supplementary material for: Impact of vaccination delay on deaths averted by pneumococcal conjugate vaccine: Modeled effects in 8 country scenarios
Source: Vaccine. 2019 Aug 23;37(36):5242–9. doi: 10.1016/j.vaccine.2019.07.063 (PMC6694201; doi:10.1016/j.vaccine.2019.07.063)
Supplement: Supplementary data 1 [file mmc1.docx]

## Supplementary Tables and Figures

Supplementary Table 1. India PCV delay scenarios under alternative schedules

| Delay | Source | #Wks Delay to 50% Final Coverage |
| --- | --- | --- |
| 3+0  Median Delay | Median delay to 50% coverage among countries on 3+0 schedule* | Schedule 6 / 10 / 14 wks  +1.5 / +2.5 / +4.5 wks |
| 3+0  Quartile Delay | Bottom quartile delay to 50% coverage among countries on 3+0 schedule* | Schedule 6 / 10 / 14 wks  +2.5 / +4.5 / +7 wks |
| 3+0 Late  Median Delay | Median delay to 50% coverage among countries on late 3+0 schedule* | Schedule 2 / 4 / 6 months  +0.5 / +1.5 / +1.5 wks |
| 3+0 Late  Quartile Delay | Bottom quartile delay to 50% coverage among countries on late 3+0 schedule* | Schedule 2 / 4 / 6 months  +2.5 / +2.5 / +2.5 wks |
| 2+1  Median Delay | Median delay to 50% coverage among countries on standard 2+1 schedule* | Schedule 2 / 4 + 12 months  +0.5 / +1.5 / +2.5 wks |
| 2+1  Quartile Delay | Bottom quartile delay to 50%coverage among countries on standard 2+1 schedule* | Schedule 2 / 4 + 12 months  +2.5 / +2.5 / +2.5 wks |
| *Among countries assessed by Clark et al (updated Dec 2014) | | |

Supplementary Figure 1. Diagram of vaccination delay model structure


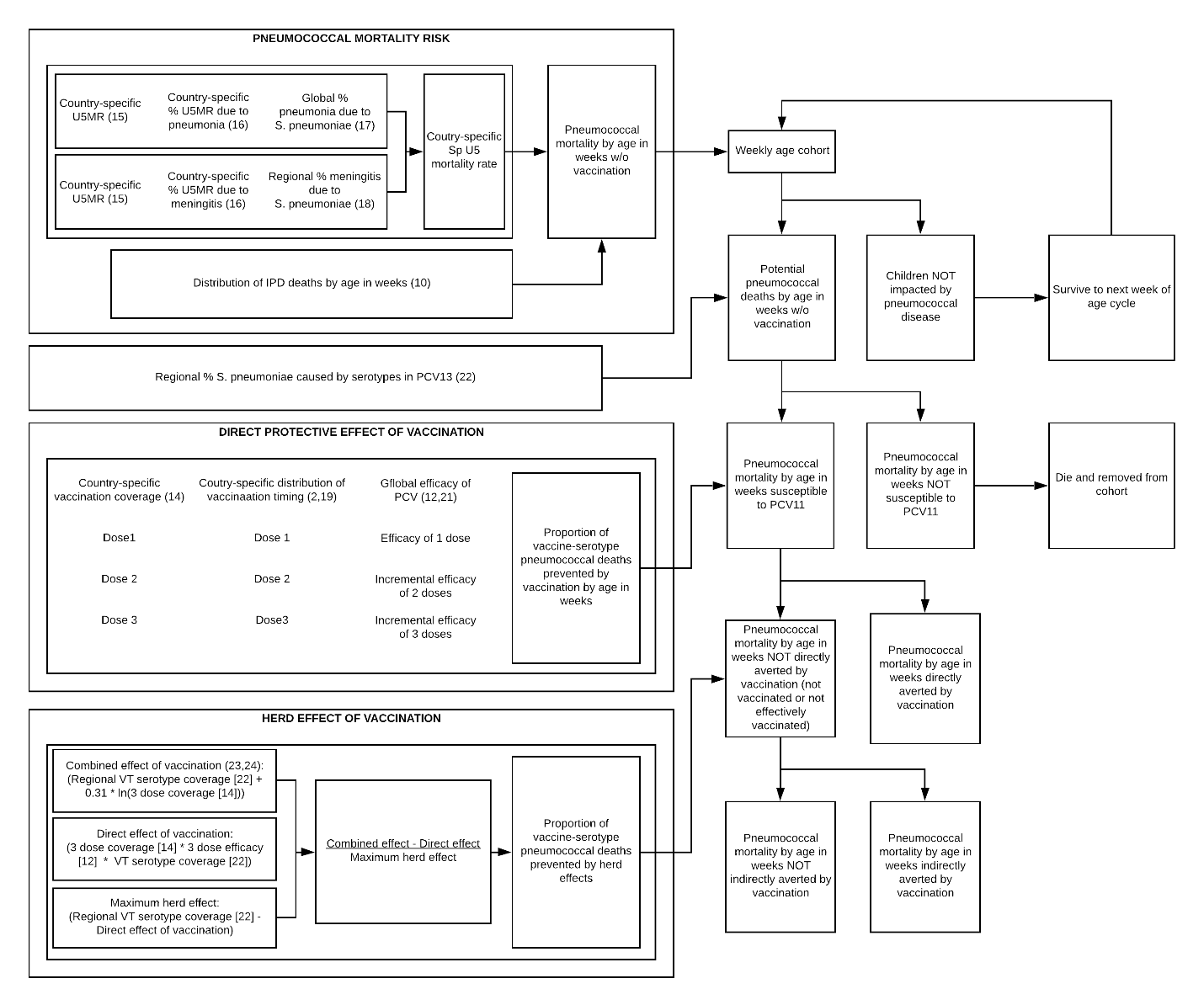


Supplementary Figure 2. Absolute and relative difference in deaths averted in India under different delay scenarios and levels of vaccination coverage, using alternative vaccination schedules
